# Arab–Israeli Leaders in Israeli Higher Education During the Iron Swords War

**DOI:** 10.3390/bs15121710

**Published:** 2025-12-10

**Authors:** Sima Zach, Mahmood Sindiani

**Affiliations:** Graduate School, Levinsky-Wingate Academic College, Campus Wingate, Netanya 4290200, Israel; mahmoods@l-w.ac.il

**Keywords:** leadership challenges, minority experiences, secondary traumatic stress, cultural bridge initiatives, proactivity

## Abstract

The study delves into the experiences of Arab–Israeli academic leaders during the Iron Swords War, revealing their emotional responses, coping mechanisms, and reflections on leadership amidst the conflict. The study highlights a range of intense negative emotions experienced by the leaders, including fear, anxiety, helplessness, and despair, reflecting a state of secondary traumatic stress. Despite facing significant challenges, some leaders demonstrated proactivity through their work duties and support from Jewish friends. However, a prevailing sense of pessimism about the future and feelings of isolation and silencing were also prominent themes in their narratives. The results underscore the complex interplay between personal experiences and the broader socio-political context, emphasizing the need for resilience-building initiatives in academia during times of crisis. The study provides valuable insights into the unique challenges faced by minority Arab academic leaders in higher education during prolonged conflict, shedding light on the importance of support systems and leadership development to navigate the complexities of wartime environments.

## 1. Introduction

Academic leadership in higher education plays a crucial role in shaping institutional vision, ensuring academic excellence, and promoting professional development. Leaders in higher education institutions are expected to possess strong administrative capabilities and a profound understanding of academic and pedagogical theories and practices (Dinh et al., 2021). Effective leadership in this context is multifaceted, encompassing academic, administrative, and interpersonal dimensions (Kezar, 2023; Lumby & Foskett, 2016). Leaders are tasked with establishing and maintaining a clear and compelling institutional vision that aligns with national educational policies and addresses the evolving needs of higher education.

When it comes to teacher education colleges, these expectations become even more specific and critical. Leaders in teacher education institutions must ensure that graduates are well-equipped to meet contemporary classroom challenges. They are responsible for creating strategic plans that translate institutional vision into actionable goals, promoting innovation in curricula, and integrating research-based practices into teacher education programs (Gigliotti & Ruben, 2017). These leaders play a pivotal role in preparing future educators by fostering a culture of academic excellence and continuous improvement (Strong, 2011).

Leaders in teacher education colleges are expected to support faculty members in engaging in scholarly research, participating in professional development, and collaborating on innovative teaching methods. This ensures that teacher education remains dynamic and responsive to emerging educational trends. Managing the complex administrative functions of these institutions is also a critical responsibility, requiring organizational skills and data-driven decision-making to maintain educational quality and institutional efficiency (Gunter, 2016).

Interpersonal skills are essential for leaders in teacher education colleges. Building strong relationships with faculty, students, policymakers, and the broader educational community fosters collaboration and institutional success (Fullan, 2007). Effective leaders must be adept at conflict resolution, communication, and team building, creating a cohesive and supportive institutional culture (Schlaerth et al., 2013).

The complexity of leading educational institutions during times of crisis, particularly from a minority position, requires a robust theoretical foundation drawing from multiple theoretical streams. This study examines three primary theoretical frameworks: Crisis Leadership Theory, Cultural Leadership Theory, and Transformative Leadership Theory.

Crisis Leadership Theory, initially developed by Pearson and Clair (1998), emerged from organizational crisis management studies but has evolved significantly in educational contexts. The theory posits that crisis leadership differs fundamentally from routine leadership in its demand for rapid decision-making, heightened situational awareness, and adaptive response capabilities. Boin’s (2009) and Boin and ‘t Hart’s (2022) subsequent work expanded this framework by introducing the concept of “facilitative crisis leadership,” emphasizing how leaders must create supportive frameworks that enable organizational resilience while managing immediate challenges.

In educational contexts, Crisis Leadership Theory has been examined extensively through studies of institutions in conflict zones (Gutman, 2024). Gutman discusses the competencies needed from educational leaders in relation to a variety of conflictual situations in eight different countries. Along the same lines as Gutman, Riggio and Newstead (2023) detailed educational leaders’ characteristics, such as sense-making in uncertainty, decision-making under pressure, crisis communication management, and organizational adaptation.

Cultural Leadership Theory, developed through the work of Walker and Dimmock (2002), provides a crucial framework for understanding leadership within specific cultural contexts. When applied to minority leaders in educational institutions, this theory helps explain how cultural identity influences leadership approaches and decision-making processes. Arar’s (2018) research on Arab educational leaders in Israel has shown how these leaders serve as cultural bridges, navigating between their institutional responsibilities and community obligations. Lord and Maher (2002) demonstrated that effective cultural leadership in education requires understanding of multiple cultural perspectives, and the ability to mediate between different cultural expectations while maintaining cultural authenticity and meeting institutional demands.

Transformative Leadership Theory, originally conceptualized by Burns (1978) and later developed in educational contexts by Shields (2010), adds another dimension to understanding leadership during crisis periods. This theory emphasizes how leaders can effect meaningful change while addressing issues of equity and social justice. In the context of minority educational leadership during crisis, Transformative Leadership Theory helps explain how leaders challenge existing power structures, maintain academic excellence while addressing social needs, create inclusive institutional environments, and build resilient educational communities.

Together, these three theories provide a comprehensive understanding of crisis leadership. They highlight the need for leaders to adjust their approaches based on the nature of the crisis, the attributes they embody, and the complexities inherent in organizational dynamics. Each theory contributes unique insights, emphasizing the significance of preparedness, adaptability, and the critical role of interpersonal relationships in effectively navigating tumultuous times.

Every crisis contains opportunities for innovation and change, and leadership feels more important now than ever. This message was conveyed by Gallos and Bolman (2021) in their recent book: *Reframing Academic Leadership*. This notion has been the foundation of many philosophers’ theories, such as those of Martin Boober (1878–1965) and Josef Schechter (1901–1994), who extensively discussed the idea that crisis is crucial to the individual spiritual and educational development (Shoshaim, 2024). A similar assertion was conveyed by MacMillan (2020), who, by displaying wars’ consequences in the history of humankind, demonstrated that civilizations made substantial progress in every aspect of life after the end of these wars. Nevertheless, using war as a “springboard” of any society demands solid inspirational leadership (Alfoqahaa & Jones, 2020). More specifically, Alfoqahaa and Jones contended that inspirational leaders such as Mahatma Gandhi, Martin Luther King, and Nelson Mandela were able to transform chaos into order. In a narrative study, the researchers revealed that these leaders were characterized by vision, non-violence, and tolerance.

Over time, there has been ongoing dialogue and examination of the ideological stances held by academic leaders throughout different historical periods. This scrutiny arises from the belief that academics play a significant role in shaping public opinions, given their engagement in activities such as research, writing, advisory positions, and consultancy. Furthermore, their direct engagement with students, who are being prepared for various specialized careers essential to the functioning of contemporary society, contributes to their potential influence (Shepherd & Shepherd, 1996).

Hence, the response of academic leaders during times of war has been a topic of interest. Shepherd and Shepherd (1996) explored the ideological orientations, particularly their attitudes towards war, of 173 honors directors in American higher education, representing (at the time of the study) the national professoriate in the U.S. as a whole. They found that college professors, especially those in social science and humanities disciplines, tend to be more liberal than conservative in their political views. They also maintained, as have others (e.g., Bulloch & Morris, 1991; Wells, 1994; Zaroulis & Sullivan, 1984), that academics have historically been prominent dissenters from government military policies during times of international conflict and war, particularly during the Vietnam War and the Persian Gulf War.

Drawing lessons from U.S. business schools during World War II, the need for innovation and adaptation to systemic challenges such as the COVID-19 pandemic, severe economic weakness, heightened inequality, racial injustice, and a climate emergency was highlighted (Tufano, 2020). The importance of strategic leadership in such times was emphasized by Barber (1992), focusing on the competencies and the effectiveness of different learning methodologies. The competencies were divided into personal characteristics, such as tolerance of uncertainty, willingness to reopen decisions, or viewing mistakes as new information, and specific capabilities such as complex cognitive skills, persuasiveness, and lateral networking.

Bernbeck (2003) discusses the challenges faced by academics in maintaining professionalism during war. His analysis illustrates how persistent issues in academic practice become more prominent during significant political crises. The shortcomings and inefficiencies of scholarly routines are underscored through examples in popularization, education, and fieldwork. As a result of these challenges, there is a need for a fundamental reconsideration of the limits of the academic sphere. These studies collectively underscore the need for academic leaders to demonstrate strategic leadership, adaptability, and a commitment to professionalism and ethical conduct during times of war.

With this in mind, we aimed to learn about the thoughts, feelings, and actions of a specific group of participants: Arab leaders in the Israeli academy during the Iron Swords War. We claim that the current research is unique and exceptional for several reasons. Firstly, it unfolds within academic institutions during a time of war, making it incomparable to any previous studies, as populations of Western countries have not experienced a wartime period for several decades. Secondly, this war began a short time after the global crisis of the COVID-19 pandemic, building upon all the lessons that were learnt regarding academic leadership in this chaotic time. Thirdly, it examines leadership during an extended period of warfare (more than five months at the time this study was written and still continuing), unlike any experienced in Israel since its establishment. Fourthly, it addresses the Arab population in Israel, which constitutes a minority, with the State of Israel engaging in war with an Arab adversary. Fifthly, it delves into a sudden, unforeseen, and prolonged extreme event that significantly impacts the entire country, including all its educational institutions and academics.

### 1.1. Research Question

How do Arab educational leaders in higher education, and specifically in teacher education colleges, experience and navigate their leadership roles during the Iron Swords War, and what strategies do they employ to maintain institutional stability and support? It should be noted that the designation “Iron Swords War” was assigned by the IDF Operations Directorate shortly after the war began. As the study was conducted during this period, participants referred to the war by this name throughout the interviews.

### 1.2. Rationale

This study addresses a critical gap in understanding how minority educational leaders navigate their complex roles during periods of acute conflict. By focusing on the experiences of Arab educational leaders during the Iron Swords War, it offers both theoretical insights and practical knowledge on crisis leadership from a minority perspective in higher education. Minority leaders often face unique challenges balancing institutional stability, community support, and professional responsibilities in times of crisis. This research sheds light on their strategies, resilience, and decision-making processes, offering insights that may benefit educational leadership in various contexts. The findings have the potential to inform policy, enhance leadership training programs, and contribute to a more inclusive framework for educational governance.

## 2. Method

This study was conducted within the interpretive paradigm, employing a hermeneutic phenomenological approach inspired by the work of Van Manen (2016). Hermeneutic phenomenology allowed the researcher to delve into the interpretations of the participants while also contributing their own insights. Hermeneutics, in this context, facilitates the interpretation of meanings and assumptions within the text that participants may find challenging to articulate, such as implicit knowledge (Altheide & Johnson, 2011; Thorne, 2016). This approach provides a framework for understanding human experiences as conveyed through language and within specific contexts (Van Manen, 2016). The goal of employing hermeneutic phenomenology was to create a vivid and compelling description of human actions, behaviors, and intentions as expressed in their narratives.

### 2.1. Participants

Ten senior Arab–Israeli academic leaders participated in the study. Four males and six females, from two teacher education colleges, two universities, one public college, one Arab college (the teaching and learning language is Arabic, and only Arab students are enrolled), the highest technological institute in Israel, and the national Israeli institute for elite sports. They all held leadership positions that will not be detailed here due to ethical issues, such as anonymity. The selection of participants followed purposive sampling criteria aimed at capturing a range of institutional and personal perspectives. We sought senior Arab–Israeli academic leaders who (a) held formal leadership or management positions in higher education institutions during the Iron Swords War, (b) had direct responsibility for staff and students, and (c) were willing to participate despite the sensitive political climate. Approximately four times as many potential participants were initially approached, but only ten consented, reflecting both the difficulty of access and the ethical sensitivity of the topic.

### 2.2. Interview

The interview was based upon the narrative approach suggested by Lieblich and Josselson (1997), conducted via the Zoom application, and lasted between 20 and 35 min. Each participant was asked to relate to one open question: “We have been in the Iron Swords War about two months; as the head of (according to their management position), and as an Arab Israeli citizen, may we, please, ask if you could tell us your authentic story, deepening as much as you can, in every aspect that you wish.” Firstly, the first author demonstrated this kind of interview to the second one, emphasizing not to add any questions, nor to lead the interviewees in any direction. Towards the end of the interview, an additional two questions were asked: “Would you like to add something else?” “Can you provide a specific situation/thought/event that has affected you the most during the last two months?”

### 2.3. Procedure and Ethical Considerations

After receiving permission from the Institutional Review Board (IRB) (#422), participants were approached via e-mail and cellular phone. A total of 37 senior academic Arab–Israeli males and females in leadership positions were approached by e-mail: 9 refused to participate, 23 did not answer, and 5 agreed. In addition, 15 were approached by phone using a text application: 7 refused, 4 did not answer, and 5 agreed to participate. The response rate was 23.8%. Interviews were conducted via the Zoom application after receiving the participants’ agreement to participate as well as to be recorded for further content analysis. We promised anonymity as well as the opportunity to regret, withdraw, and even delete things that they say (which happened with one of the participants who wanted to firmly hold anonymity).

Beyond formal approval and informed consent, additional safeguards were implemented to protect participants from deductive disclosure and potential professional or political risks. All institutional and geographic identifiers were removed or generalized, and participants reviewed their transcripts to approve or delete any sensitive content. Interviews were conducted through secure, password-protected Zoom sessions, with recordings stored on encrypted, access-restricted drives. Communication was maintained via personal rather than institutional channels. These measures ensured confidentiality and participant safety under the sensitive socio-political circumstances of the Iron Swords War.

### 2.4. Data Analysis Transparency and Trustworthiness

The analytic process followed a systematic hermeneutic–phenomenological procedure (Van Manen, 2016; Titchen & McIntyre, 1993). After verbatim transcription of each interview, both authors independently conducted line-by-line open coding to identify preliminary meanings. These raw descriptive codes were then grouped into first-order categories (participants’ explicit statements) and second-order categories (interpretive researcher constructs). Following several iterative meetings, discrepancies in coding were discussed until full consensus was reached through negotiated interpretation rather than statistical agreement, ensuring conceptual coherence (Brink, 1993).

Thematic saturation was established when no new categories emerged after the ninth and tenth interviews, following the criteria proposed by Hennink and Kaiser (2022). Given the sensitive political context and a 23.8% response rate, we recognize the potential for self-selection bias: participants who felt safer or more reflective may be over-represented, while those experiencing greater fear or institutional pressure might have refrained from participation. This limitation does not undermine the study’s credibility but does constrain transferability, as the findings portray the perspectives of those willing to speak within a tense socio-political environment. Reflexivity and contextual description are therefore used to support readers’ judgment regarding the applicability of results to other minority or conflict settings.

It is worth noting that the second author conducted most of the interviews because he belongs, like the participants in the study, to the Arab society. In this way, we addressed the issue of reflexivity and a higher level of trust or openness among the respondents. One of the participants emphasized that if the interviewer was not the second author, he would not have participated in the study. Narrative data analysis does not simply involve understanding the content but tries to interpret the particular cultural context (Riessman, 2012). The participants’ stories require further analysis to cluster data under themes or patterns, allowing idiosyncratic findings about the phenomenon to emerge. From Riessman’s (2012) narrative analysis typology that identifies four models of narrative analysis, we used a combination of the thematic analysis, which emphasizes what is said, and the structural analysis, which emphasizes the way the story is told. The thematic approach is useful for finding common elements across the participants’ experiences, while in the structural model, the focus moves to the way the story is told.

## 3. Results

**Figure 1:**
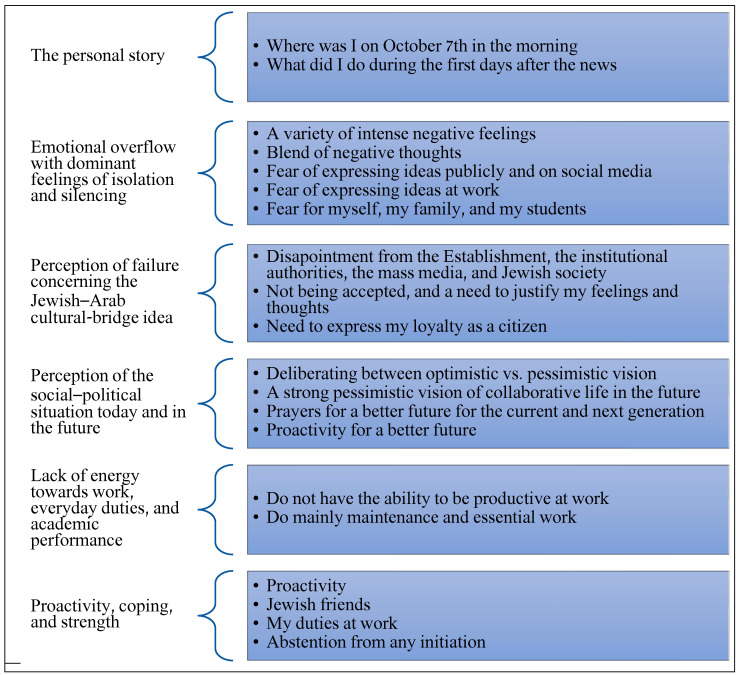
First- and second-order categories of content sorted by themes that emerged from the interviews.

Data analysis revealed several main themes that emerged from the participants’ monologues. These themes were then grouped by both authors into six topics: (1) the personal story—how the events of October 7 affected them personally; (2) emotional overflow, including feelings of isolation and silencing, (3) critique/perception of failure regarding the Jewish–Arab cultural bridge idea, (4) perception of the social–political situation today and in the future, (5) lack of energy towards work, everyday duties, and academic performance, and (6) coping and strength resources (see Figure 1).

The following are examples of quotes that were given by the participants for each of the themes and sub-themes that emerged from the analysis.

1. **The personal story—how the events of 7 October affected me personally.** There are several natural disasters and manmade incidents or events that everyone who was living on Earth while they occurred will remember for the rest of their lives; specifically, they will remember not only what happened, but also where they were, and what they were doing (such as the President Kennedy assassination, the 9/11 Twin Towers collapse, Prime Minister Yitzchak Rabin’s assassination, the beginning of the 1973 Yom Kippur War in Israel, the UN declaration establishing Israel as a State for the Jewish Nation, etc.) (e.g., Monteil et al., 2020; Russo & Moses, 2013). This was evident in our interviews. All the participants, without exception, at the onset of the interview chose to describe where they were and what they were doing at 06:30 AM. 7 October was several days before the beginning of the new academic year. Hence, many people were still on vacation abroad. Half of the participants were abroad and described in detail what they did from the moment they heard the news to the moment of their return home. Here are several examples:

“I was not in the country. Immediately as I heard the news, I tried to purchase the tickets back home. It was chaos, almost all airlines canceled their flights to Israel due to the war that began. I had to travel through two airports in Europe, and it took 48 h to return.”

(6)

“It was on Saturday early morning, while people were still at home. I was on the second floor at home. I rushed down and turned on the TV. The news spread out very quickly, all day long and ever since, but understanding the event took hours, days, and weeks”.

(2)

2. **Emotional overflow.** At the beginning of the October 7th war, social media was a major news supplier. Films arrived through all existing channels—Telegram, Instagram, Facebook, TikTok, and the mobile channels of the TV and radio, both from the Hammams and from the Israeli soldiers and civilians that experienced the horrors.

“The enormity of the horror was beyond understanding. Most of all, we could not believe that the strong IDF was totally surprised and defeated. We really could not believe what we saw and heard. As a religious Muslim, I must say that I was ashamed, and even offended. Such inhuman brutality does not exist in our religion. I cannot imagine what those who planned the attack thought they were doing. I immediately knew the consequences, and I was devastated.”

(7)

“I was totally shocked. I couldn’t believe what I heard and saw. I tried not to look at films at all! How people take other people’s lives. It is against all our beliefs. I was so scared of what was going to be next. I cared for myself and my family, especially the kids. I knew that revenge would be huge and thousands of people would pay.”

(3)

“In the first several weeks after the war started, I was occupied with my students. I felt that I must be there for them. Some were very scared to go out, to take public transportation, even to come to the campus. Some of my students are young and have never experienced such a chaotic situation. I felt that I had to take care and instruct them, but I was not exactly sure how to do that.”

(5)

**Feeling of isolation and silencing**. From the response rate, one can conclude that more than 70% of the potential participants perceived the aim of the study as complex and highly sensitive, which led them to recoil and be unwilling to openly share their thoughts and feelings. Nevertheless, hard feelings were vigorously expressed, as described in the following examples:

“As an individual belonging to the Arab society minority, I felt isolated within the academic world. I felt silenced, so I deliberately avoided speaking or talking publicly and on social media, at least in the first several weeks. I felt that I had to justify myself, to declare again my loyalty to the Jewish society, as if I were not a citizen with equal rights. Actually, these feelings arose doubts regarding my or our place (the Arab society) in the general society.”

(2)

“We are not only excluded, but we exclude ourselves and give up our right to express our opinion, because on that we may pay a heavy price.”

(8)

“I wanted to discuss the situation with my students, but I did not dare. I was afraid to deal with the situation since the media was full of examples that Arab students and faculty were brought before discipline committees on the grounds of incitement and many were warned, expelled, suspended or even fired.”

(10)

“Initially, I hesitated to express my opinions. Despite being known for my outspoken nature, I chose silence, opting instead to observe and absorb my surroundings. Reacting without a clear understanding of those around you is unwise. When I finally spoke, I declared, “You know nothing about me, yet I know everything about you.” This declaration was met with silence and restraint. With each passing moment of quiet reflection, my sense of self solidified, elevating my contemplation of self-identity to a higher level.”

(3)

“It hurt me immediately and immensely; I never wished for us to find ourselves in the situation we faced on October 7th. I even feared it jeopardized the rights and prospects of the Palestinian people. In the aftermath of the attack, my mind was consumed with questions about the path ahead. Where would it lead us? What fate awaits my children? Will they depart our homeland, only to return later? Perhaps I’ll be compelled to start anew elsewhere. I cannot endure residing in a place where speech is stifled, where expressing oneself freely is restricted. I feel utterly powerless, overwhelmed by suffering.”

(6)

“I found myself unable to freely express my thoughts in various forums. I grappled with questions about my identity: am I Israeli, Arab, Palestinian? Could a significant transformation occur? My mind swirled with these thoughts. I struggled with the dual anguish of the events of October 7th and the ongoing turmoil in Gaza, all while residing in Israel and striving to maintain my current lifestyle. What provided me strength were my Jewish friends. Their support was invaluable, even amidst our disagreements.”

(9)

3. **Critique/perception of failure regarding the Jewish–Arab cultural bridge idea.** Although they were not surprised, interviewees, without exception, expressed their disappointment with “the Establishment”. They emphasized that their belief in coexistence between the Arab–Israeli society and the Jewish–Israeli society was badly and negatively affected. The memories from Operation Guardian of the Walls that occurred only three years earlier were still fresh. In that operation, according to the State Comptroller’s report, three civilians were killed, and hundreds were injured (including approximately 306 police officers) in approximately 520 documented incidents, at the peak of which, according to the assessment of authorized security officials, approximately 6000 Israeli–Arab residents participated. About 3200 people were arrested, including about 240 Jews (State Comptroller Report, 2022.07.27). Some said that they felt again the need to express their loyalty as a citizen, as follows:

“Every time that the Israeli society suffers an Arab attack of any kind and scale, we, as Arab–Israeli citizens, have to express our loyalty. It is expected of us to condemn initiation of hatred, violence, and terror. As if one can accept such behavior. It is against my belief to take other people’s lives. It is also against our religion. Still, it is expected that I will say it or write about it clearly.”

(9)

“As an Arab, I could not feel part of the whole society; the divide between the two societies was immediately present. I had to justify my feelings and thoughts. I have family in Gaza, and I knew at the onset of that horrible morning that painful revenge would come. I felt devastated.”

(10)

“I felt disappointment from the Establishment, the institutional authorities, the mass media and the Jewish society. Suddenly, the whole perception and hope for coexistence sound to me ironic and hypocritical.”

(5)

“As the weeks passed, I became more confident, and I raised the voice of disappointment from the Jewish society.”

(8)

4. **Perception of the social–political situation today and in the future.** The social–political situation in Israel since it became an independent country is fragile and has known constant difficulties (Sela, 2012). These difficulties are rooted in the history of both nations that live in the same country (Farsakh, 2011). Since the declaration of its independence, Israel has had to manage seven wars, dozens of operations, and thousands of terror attacks, which have been part of its population’s existence. Since wars and operations are a part of life, all Israeli citizens at the age of 18 are recruited to mandatory service in the army, while the Arab–Israeli citizens can choose to serve in the “civil service” (Amidror, 2018). Israeli Arabs exist as a minority within Israeli society, with the intricacies of their daily lives posing challenges to their connection to the State of Israel and their sense of inclusion within the broader societal fabric (Jonas & Fritsche, 2013). While officially recognized as citizens with voting rights and legal equality, they concurrently grapple with a complex dual identity (Levy et al., 2017; Pilecki & Hammack, 2014). They are actively engaged in the nation’s public institutions, yet their bonds often extend beyond Israel’s borders to include kin in areas such as Judea and Samaria, the Gaza Strip, and neighboring Arab nations. During periods of conflict, military campaigns, and acts of terrorism targeting Israel and its populace, this dual allegiance intensifies, as illustrated by the following excerpts:

“At the moment that I heard the horrible news, I knew that it would have a detrimental effect on the whole region for years. I see at least ten years of wars from now. All the fragile achievements that we have achieved will disappear. That was immediately very clear to me.”

(1)

“On the one hand, I do not see hope for me or for my children; on the other hand, I am still optimistic, and I think that if we invest and are proactive toward our coexistence in this country, someday we will be able to live here together peacefully.”

(4)

“I had a hope that a change would occur and that I would be part of the change, but the hope goes further away as years go by.”

(10)

“I decided that unless I will to be proactive, I will never be able to come up with complaints to anyone. I think that we constantly and usually cry more about the situation than being proactive towards real and positive change.”

(6)

5. **Lack of energy towards work, everyday duties, and academic performance.**

We were purposefully interested in surveying the population of Arab academic leaders. Relying on Bernbeck (2003), who discusses the challenges faced by academics in maintaining professionalism during war, we searched for the unique voices of minority leadership. In addition, following Shepherd and Shepherd (1996) and others (e.g., Bulloch & Morris, 1991; Wells, 1994; Zaroulis & Sullivan, 1984), we were interested to find out whether their ideological orientation and political views would be expressed. The following are some examples representing their activities and orientations regarding their academic position:

“I maintained office duties, working very hard 24/7 to give support and help with every problem that arose. There were endless problems”.

(7)

“I felt sorry for my students who were eager to talk and get some relief from the high stress that they felt. Nevertheless, I could not afford it, because I understood that talking is forbidden. Hence, we continued with teaching routines.”

(2)

“I completely left anything that is related to writing. Research is a luxury in such a chaotic time. Nothing. Nothing at all! In addition, I think that it would affect Arab researchers. They will receive less grants if they will receive any.”

(3)

6. **Coping and strength**

Since the Iron Swords War has lasted for months (and is still continuing), the need for coping resources goes without saying. We expected this issue to be present in the participants’ narratives, but the focus was less on strengths and more on weaknesses, hopelessness, helplessness, and even despair, as we presented above. Nevertheless, four aspects were mentioned regarding coping resources: (1) proactivity, (2) Jewish friends’ support, (3) staying occupied with duties at work, and (4) abstention from any initiation, as described in the following quotes:

Proactivity: “At the beginning, I helped to translate conversations that were spread on social media due to winds of violence that were expressed in both sides, Arabs and Jewish. Then, I decided to express my opinion, and I refused to be a translator. I thought that I had much more important duties as an educational leader. Talking was the first important step.”

(5)

“Due to the war, I began to be politically involved. I started to search for organizations. I felt the need and I recognized my ability to lead to a better place. To give my part to coexistence.”

(7)

“I felt a sense of duty, utilizing every opportunity to learn more about Arab society in Israel. I recognized the necessity of striking a balance; coexistence is imperative, not optional. It’s the reality we’re faced with, and I believe we can strive for a fulfilling life within it.”

Jewish friends: “What really comforted me is my Jewish friends from Israel and from the USA who called to support and ask how am I during this war and uncertain time. It is really comforting that I have a circle of people and friends that can understand the complexity of the situation that we encounter. It is meaningful to me and keeps me optimistic.”

Duties at work: “I am busy 24/7 with lots of duties. I volunteer to take more and more duties. Being highly active keeps me sane.”

Abstention: “In this war, the Arab–Israeli society chose an attitude of abstention. I think it sends a message which is kind of loyalty, but at the same time, we could not be more involved due to what we see happening in Gaza. You know, between the Palestinians and the Jewish Israelis, there is disconnection, and even blindness and impermeability to the pain of the other. The Israelis do not see the Palestinians and vice versa, and we, the 48’ Arabs who live here and see both sides and understand both sides, are stuck between the hammer and the anvil.”

## 4. Discussion

The experiences of Arab–Israeli academic leaders during the Iron Swords War reveal a complex interplay of emotional responses, coping mechanisms, and leadership strategies, all of which can be framed within established theoretical frameworks of crisis leadership, cultural leadership, and transformative leadership. This discussion examines how these theories illuminate the findings derived from the participants’ narratives, particularly as represented in Figure 1’s main themes, which outline their personal stories, emotional burdens, and perceptions of isolation, cultural bridging, and coping resources.

Central to understanding the participants’ emotional experiences is Crisis Leadership Theory, which posits that effective leadership during times of crisis necessitates rapid decision-making, heightened situational awareness, and adaptive responses (Pearson & Clair, 1998). The intense emotions articulated by the academic leaders, such as fear, anxiety, and despair, reflect the psychological toll of navigating leadership roles during a protracted conflict. The concept of secondary traumatic stress emerges prominently here, as leaders contended with their personal experiences of war while simultaneously managing institutional responsibilities. The narrative data illuminated a widespread feeling of helplessness and a critique of their perceived effectiveness in fostering communication across cultural divides. This aligns with Boin and ‘t Hart’s (2022) expansion of Crisis Leadership Theory, emphasizing the need for leaders to establish supportive frameworks that not only address immediate organizational challenges but also buffer against the psychological impacts of crisis.

Within the context of the emotional overflow reported by participants, it becomes evident that the disconnection between Jewish and Arab communities exacerbated feelings of isolation and silencing. The leaders’ narratives expressively illustrated their struggle to serve as cultural bridges amidst prevailing tensions, casting doubt on the effectiveness of existing intercultural initiatives aimed at fostering coexistence. As noted by Gutman (2024), successful crisis leadership in educational contexts necessitates an understanding of the unique dynamics at play in conflict situations. The leaders’ expressions of feeling trapped between two conflicting narratives, while desiring peace and understanding, resonate deeply with the challenges outlined in Crisis Leadership Theory, underscoring the complex relationship between institutional stability and the emotional well-being of leaders. A big question that remained unsolved is the real reason for those feelings. Since no one further explained, we can only conclude with caution that the magnitude of the terror attack may have led some participants to envision worst-case scenarios concerning its possible consequences, a psychological process that warrants further exploration in future research.

Disappointment with the Jewish–Arab cultural bridge idea was extensively expressed. As presented, the majority of the emotions were deeply and intensely negative. While, objectively, war and the reason for it are devastating, the questions that arise from these results, which have no answer, and should be examined in follow-up research, were the following: Why did responsible Arab academic leadership fail to act when action was most critical? Why did they feel so paralyzed for the first few months of the war? How come the vision of these leaders is generally more pessimistic than optimistic? It certainly highlights the importance of re-examining the academic leadership vision, mission, and goals.

Cultural Leadership Theory further enhances our understanding of the participants’ experiences by highlighting the significance of fostering a cohesive institutional culture, particularly in times of upheaval (Walker & Dimmock, 2002). The emotional narratives revealed not only the personal burdens these leaders carried but also a profound sense of responsibility toward their institutions and communities. Some of the participants engaged in proactive behaviors, such as assuming additional duties and actively seeking support from colleagues, in an effort to maintain stability and promote a sense of belonging among faculty, staff, and students. This aligns with Schlaerth et al.’s (2013) assertion that effective leaders cultivate collaboration and a supportive environment.

The leaders’ commitment to their professional roles amidst chaos reflects the tenets of Cultural Leadership Theory, where a leader’s ability to understand and embrace cultural dynamics becomes crucial in fostering resilience within their institutions. Participants expressed a desire to balance an acknowledgment of the crisis’s emotional fallout with an ongoing commitment to coexistence. This dual focus speaks to the potential of leaders to facilitate meaningful dialogue and connection even in a polarized environment. Additionally, the narratives indicated a collective desire for cultural understanding that transcended individual experiences, further emphasizing the need for culturally informed leadership approaches during times of conflict.

At the same time, the findings indicate that the leaders’ experiences were marked by significant barriers to effective intercultural bridging. The dissatisfaction with previous cultural initiatives suggests the need for critical reflection on the efficacy of such programs and the potential for deeper engagement with minority perspectives. As highlighted by Alfoqahaa and Jones (2020), the ability to turn crises into opportunities for growth requires strength of leadership capable of navigating the complexities of intergroup relations. The desire shared by our participants for constructive dialogue and visible support from Jewish colleagues suggests an urgent need for inclusive practices that empower minority voices while fostering a culture of collaboration.

Transformative Leadership Theory offers another lens for interpreting these experiences. This theory emphasizes the capacity of leaders to inspire change and foster resilience in times of adversity (Shoshaim, 2024). The transformative elements revealed in participants’ engagement with their responsibilities, where their proactive responses served as a source of sanity amid chaos, underscore the essence of leadership as a transformative process. The initiatives taken by leaders to be more involved in their roles, despite emotional burdens, embody the transformational qualities that can reshape the narrative surrounding minority leadership during wartime.

The themes of coping and strength resources represented discoveries of personal agency within oppressive circumstances. Our participants found solace in their work, viewing it as a vital avenue for maintaining stability within their institutions. This finding reflects insights from Bernbeck (2003), who underscores the necessity for academics to demonstrate professionalism and adaptability during crises. Actively participating in academic responsibilities not only gave these leaders a sense of purpose but also strengthened their determination to serve as community leaders during challenging times. Thus, their narratives illustrate how acts of agency can catalyze transformative outcomes, even amidst overwhelming challenges.

Empowerment and delegation of authority were not expressed in any of the interviews. The omission/silencing/absence of a prominent aspect within the main examined subject reflects something that the interpreter should relate to (Spector-Mersel, 2008, 2010). Why did no one talk about the empowerment of the people they were in charge of? Why did no one talk about the delegation of authority to others? The absence of references to empowerment or delegation among participants may suggest contextual constraints on leadership expression. This raises an open question about how fear or institutional pressures shape leadership responsibility during prolonged crises. While this study provides valuable insights into the narratives of Arab–Israeli academic leaders, it is important to acknowledge certain limitations. First, the rate of people who were eligible to participate and refused, or did not respond to our invitation, was quite high. Therefore, we should consider the results with caution. Still, a sample size of ten interviews provided saturation to the stories that were told, indicating this is a satisfactory number of interviewees for this qualitative study (Hennink & Kaiser, 2022). Also, it should be noted that a half-hour interview may not cover all the essential aspects of leadership in the context of war. However, given the participants’ demanding work schedules, securing a longer interview was nearly impossible. In addition, leadership can be tested in the short term, but we should keep in mind that leadership is also a process. Since the war is still continuing while we are writing this article, examining the process that these leaders went through might give a broader understanding of their functioning.

**Figure 2:**
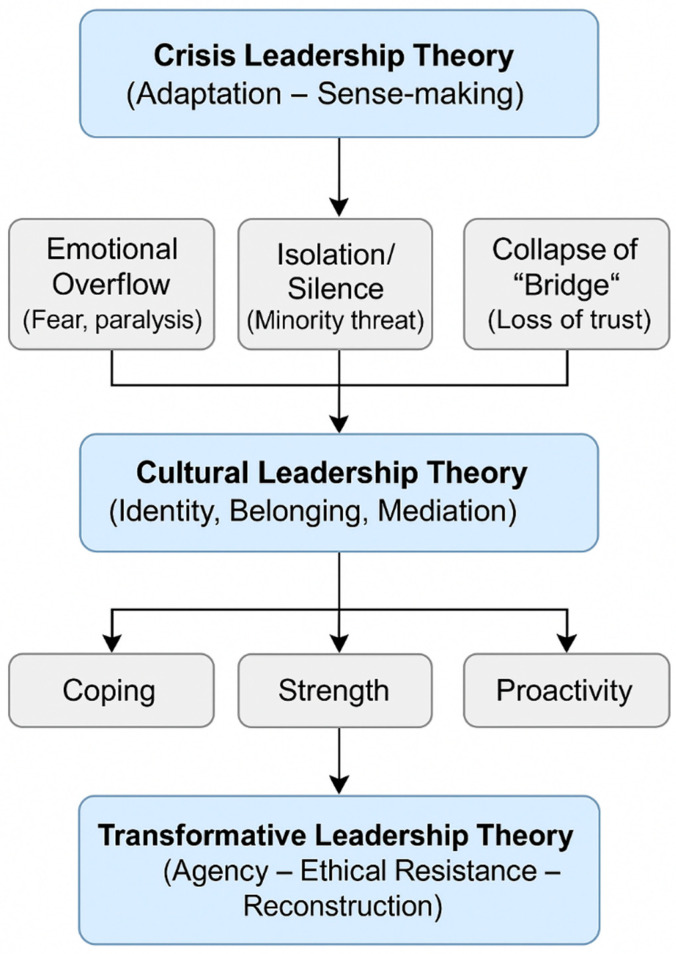
Integrative infographic.

Figure 2 clarifies how the study’s findings confirm, refine, and extend the three leadership theories.

Figure 2 visualizes the flow from *crisis response* (crisis leadership) through *cultural mediation and identity negotiation* (cultural leadership) toward *ethical and transformational agency* (transformative leadership). It portrays leadership not as a static construct but as an evolving process, from emotional shock to reflective action and finally to reconstructive hope.

This depiction emphasizes how minority academic leaders transition from survival to meaning-making and institutional resilience, thereby enriching all three theoretical frameworks.

### The Practical Implication

The findings of this study have several practical implications for academia, leadership, and conflict resolution. First is resilience-building, which, as we have seen, is needed during times of extreme crises such as war, in the academic leadership arena. While some participants demonstrated resilience and proactivity in their personal lives, there is an opportunity to support and amplify these efforts at the institutional level. Academic institutions can invest in resilience-building programs, leadership development initiatives, and community-building activities to foster a sense of agency and empowerment among faculty, staff, and students.

Second, the absence of proactive leadership behavior during the crisis highlights the need for inclusive leadership practices that empower individuals at all levels of the organization. Academic leaders should be able to foster a culture of open communication, collaboration, and shared decision-making to ensure that diverse voices are heard and valued. Lastly, reevaluation of cultural bridge initiatives: The disappointment expressed by participants regarding cultural bridge initiatives suggests a need for critical reflection and reevaluation of intercultural dialogue and reconciliation efforts. Institutions and policymakers should engage in meaningful dialogue with minority communities to better understand their perspectives and address underlying tensions and grievances. Lastly, long-term conflict-resolution strategies: This study highlights the challenges of leadership in ongoing conflict situations and the need for sustained, comprehensive conflict-resolution strategies. Academic institutions, policymakers, and community leaders should work collaboratively to address underlying socio-political issues and create inclusive environments conducive to peacebuilding and reconciliation.

## 5. Conclusions

This study illuminated the multifaceted experiences of Arab–Israeli academic leaders during the Iron Swords War, revealing the profound emotional, cultural, and ethical challenges of leading from a minority position amid national crises. Their narratives exposed intense fear, isolation, and moral conflict, yet also moments of resilience, proactivity, and solidarity. The findings highlight the intersection of Crisis Leadership, Cultural Leadership, and Transformative Leadership Theories: leaders faced acute uncertainty and secondary traumatic stress while striving to sustain institutional stability and moral integrity. The perceived collapse of the “cultural bridge” between Jewish and Arab communities underscored the fragility of coexistence narratives in times of war. Nonetheless, acts of engagement, such as continuing academic duties, supporting students, and fostering dialogue, demonstrated leadership as a form of resistance and reconstruction.

These insights underscore the urgent need for higher education institutions to develop inclusive leadership frameworks that integrate crisis preparedness, resilience training, and intergroup dialogue. Future research should longitudinally explore how minority leaders’ identities and strategies evolve beyond wartime contexts. Ultimately, leadership in crisis is not only administrative but profoundly human, anchored in empathy, cultural awareness, and the capacity to transform despair into renewed commitment to coexistence and peace.

## Data Availability

Data is unavailable due to privacy and ethical restrictions.
